# Comparison of a Multi-Scenario Robustness Evaluation Method with Measurements for Proton Teletherapy †

**DOI:** 10.3390/cancers17172927

**Published:** 2025-09-06

**Authors:** Qiangxing Yang, Michael F. Moyers, Zhuangming Shen

**Affiliations:** 1 Department of Medical Physics, Shanghai Proton and Heavy Ion Center, Fudan University Cancer Hospital, Shanghai 201315, China; 1521009@zju.edu.cn; 2 Shanghai Key Laboratory of Radiation Oncology, Shanghai 201315, China; zhuangming.shen@sphic.org.cn; 3 Shanghai Engineering Research Center of Proton and Heavy Ion Radiation Therapy, Shanghai 201315, China; 4 Department of Medical Physics, Shanghai Proton and Heavy Ion Center, Shanghai 201315, China

**Keywords:** proton, radiotherapy, uncertainty, robustness, multi-scenario

## Abstract

*Background/Objectives*: Multi-scenario calculational methods have been used to evaluate proton teletherapy plan robustness but few studies have been performed to determine the accuracy of these calculational methods. This study evaluates a multi-scenario method by comparing calculations to measurements made in phantoms that simulate the effects of possible uncertainties. *Methods*: Plans were made using four phantoms in which the delivered dose was highly sensitive to positional and penetration uncertainties. The effects of alignment and penetration uncertainties on the dose distributions of each of those phantoms were simulated by performing calculations using nine different uncertainty scenarios and comparing the calculations to measurements with induced physical alignment displacements. Measured dose distributions were obtained by exposing films placed inside the phantoms and extracting multiple linear profiles. The maximum and minimum doses obtained for each of the calculational scenarios were compared with the measured dose profiles. In addition, comparisons of DVHs for nominal and uncertainty scenarios were performed. *Results*: The results showed that, under the influence of uncertainties, the minimum dose for the four phantoms decreased by more than 20 Gy, the V_95%_ coverage fluctuated by more than 10%, but the maximum dose parameter changed by less than 5 Gy. This was expected, as no margins for uncertainties were applied around the targets. The envelope bounded by the maximum and minimum possible calculated doses contained most of the measurements, although the shapes of the dose profiles displayed some mismatches for wedge and head phantoms. There were a few points where the measured maximum dose for bone and lung slab phantom cases was slightly higher than the maximum dose calculated from the nine scenarios. *Conclusions*: This study demonstrates that a nine-scenario method can adequately evaluate the robustness of simple mono-directional plans containing heterogeneities.

## Simple Summary

There are many uncertainties in the planning and delivery of treatment with radiation, especially when light ion beams are used. Calculational methods to estimate possible delivered dose distributions have been proposed and implemented but seldom have the results of these calculations been compared to measurements. This study compares a multi-scenario method to dose measurements made in several phantoms using various scenarios.

## 1. Introduction

Compared to photon teletherapy, proton teletherapy can provide a higher dose to a target while reducing the dose to organs at risk (OARs). This is due primarily to the proton’s finite depth of penetration and the inverse dose distribution with depth, i.e., the Bragg peak. There exists, however, uncertainties about the lateral alignment of the patient and the proton’s depth of penetration. Accounting for these uncertainties is an essential aspect of planning for radiotherapy to prevent patient harm and make an effective treatment. Many proton beam facilities still use the planning volume target (PTV) concept, a method used in X-ray therapy, to estimate the margin required to mitigate the effect of potential uncertainties. In the PTV concept, the treatment plan can be regarded as creating a static dose cloud independent of the shape of the patient (including heterogeneities) [1,2]. In proton teletherapy, however, the difference in penetration depth for different proximal water equivalent thicknesses, either from day-to-day anatomical variations or lateral alignment uncertainties between the beam and the patient, makes the static dose cloud hypothesis invalid, leading to the ineffectiveness of PTV-based planning [3,4,5]. This makes the evaluation of uncertainties for proton and other ion teletherapy more important than for X-ray therapy. For proton teletherapy, planning with robust methods is considered a superior approach to planning with PTVs [6].

Recently, some commercial Treatment Planning Systems (TPSs) have implemented various methods to generate plans that satisfy the prescription when the treatment is off-nominal but still within the bounds of possible uncertainties; these methods have been called “robust planning”. The plans resulting from the application of these methods must still, however, be assessed to analyze the extent of robustness. The main two types of uncertainties to be considered in evaluating the robustness of a plan are patient alignment with respect to the beam and beam penetration [7]. A survey of uncertainties in light ion beam therapy has been published by the American Association of Physicists in Medicine (AAPM) Task Group 202 [8]. An early method of estimating the uncertainties was provided by Goitein, which calculated three different dose distributions by expanding and contracting aperture and bolus shapes [9]. This method is difficult, however, to apply to energy and fluence modulated ion beams. An alternative method is to calculate and simulate a variety of dose distributions with different errors, a so-called “multi-scenario” method [10]. In a typical multi-scenario method, scenarios are calculated that include the over-penetration and under-penetration of the protons to account for uncertainties such as XCT numbers, converting XCT numbers to a relative linear stopping power, beam energy, and the thickness of beam modifiers such as boluses and tabletops. Alignment uncertainties are also simulated to account for items such as gantry sag, spot position, spot size, target contouring, and X-ray localization.

Korevaar et al. used a multi-scenario evaluation method that is applicable to both photon and proton treatments and was seen to be consistent with historical PTV-based treatment plan evaluations [2]. In the study of Wei et al., a worst-case dose distribution for evaluation was generated using a nine-scenario method. The result shows that a worst-case optimization method yielded a plan that was considerably less sensitive to range and setup uncertainties than a PTV optimization method [6]. Sterpin et al. evaluated several robustness evaluation methods for proton treatment plans. They compared 20, 80, 240, and 1000 scenarios and observed that the width of DVH bands and the confidence levels of robustness depended upon the method chosen to sample the scenarios [11]. Noufal et al. created 16 scenarios containing both setup and penetration uncertainties and studied the perturbations of the nominal plan dose distributions. They concluded that a robustness evaluation should be considered as a part of the normal plan evaluation procedure, even for X-ray plans [12]. Yock described a 13-scenario method wherein a translational setup error of ±5 mm in the left–right, anterior–posterior, or superior–inferior directions was combined with a ±2% penetration uncertainty. They explained that robustness analyses should be promoted to yield reliable plan evaluations and dose reporting, particularly during clinical trials conducted by multiple institutions using multiple treatment modalities [13]. Rana et al. evaluated the impact of spot size on plan robustness for lung cancer plans in pencil beam scanning proton therapy. In their study, plan robustness was investigated for 12 perturbed scenarios, which combined isocenter shifts with penetration uncertainty. They concluded that use of a small scanning spot resulted in CTV coverage that was more robust against setup and penetration errors compared to the use of a large spot [14]. Ödén et al. presented a method of incorporating the variable RBE and its uncertainties into the proton plan robustness evaluation [15]. They also calculated 81 error scenarios to analyze the effect of the increase in relative biological effectiveness (RBE) near the Bragg peak. They concluded that, although a direct causality between RBE and toxicity could not be established, a high linear energy transfer (LET) and RBE-weighted dose (D_RBE_) were correlated spatially with the observed toxicities, whereas setup and penetration uncertainties had a minor impact [16].

Most of the above studies concerning an uncertainty analysis were performed without performing measurements to verify the calculational methods. In this study, a nine-scenario method was compared with measurements in phantoms to assess the method’s ability to predict maximum and minimum possible doses at each voxel within a patient.

## 2. Materials and Methods

### 2.1. Phantom Planning

**Figure 1:**
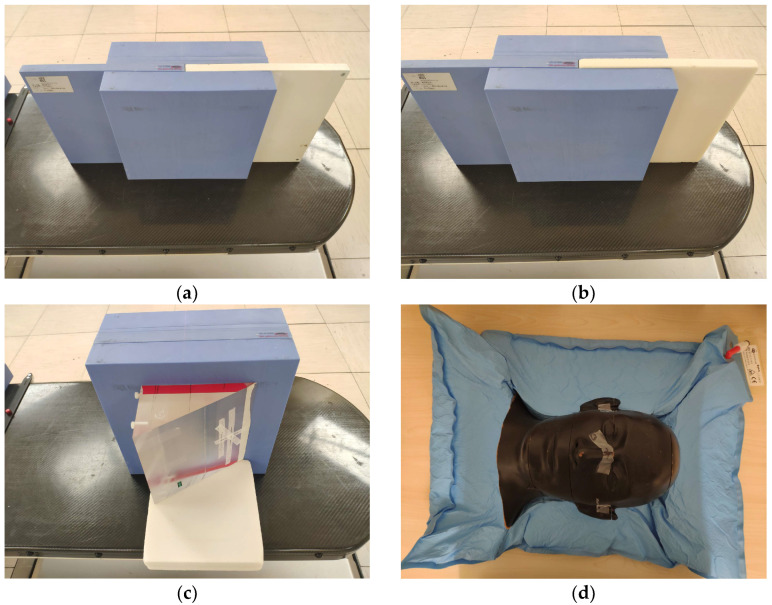
Photographs of the four phantoms. (**a**) Bone and solid water phantom combination. (**b**) Lung and solid water combination. (**c**) PMMA wedge and solid water combination. (**d**) Rando head phantom.

To test the multi-scenario method of robustness evaluation, four phantoms were chosen for which the delivered dose would be highly sensitive to positional and penetration errors. Figure 1 shows photographs of the four phantoms. The first phantom, seen in Figure 1a, consisted of a slab of bone-equivalent plastic (450 CTG, Gammex, Middleton, WI, USA) sandwiched between slabs of water-equivalent plastic (557 CTG, Gammex, Middleton, WI, USA), hereafter referred to as solid water. This phantom allowed testing of a relatively high-density, high-atomic-number material. The second phantom, seen in Figure 1b, was similar in composition but used a slab of lung-equivalent plastic (455, Gammex, Middleton, WI, USA) instead of bone. This phantom allowed for the testing of low-average-density material. The third phantom, seen in Figure 1c, consisted of a polymethylmethacrylate (PMMA) wedge placed in front of the solid water. The wedge was fixed in front of the water phantom so that at a given depth within the solid water phantom, the ions stopped near the distal side of the target. The fourth phantom, seen in Figure 1d, was a copy of a phantom previously used in space radiation experiments (Rando, The Phantom Laboratory, Greenwich, NY, USA) and was divided into slices of various thickness, with some slices containing holes to insert radiation detectors [17,18,19]. For this study, only the head of the phantom was used. The internal structure of the phantom included real human bones, thus providing a case that is closer to the actual treatment.

**Figure 2:**
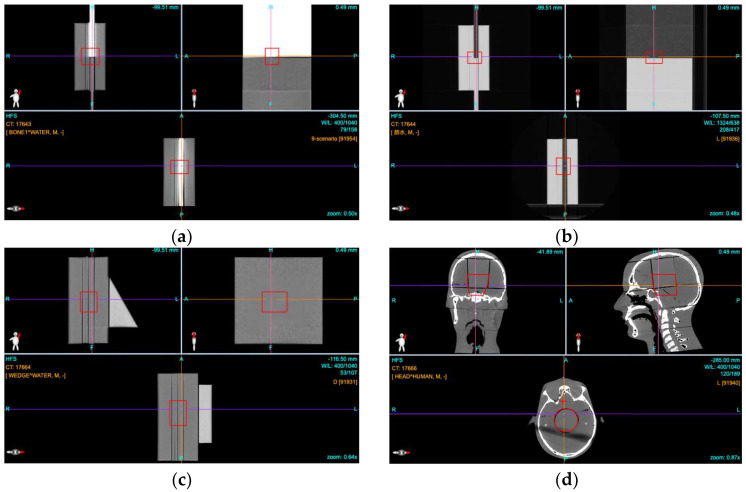
The targets for each of the four phantoms are shown by red contours. (**a**) Bone and solid water phantom combination. (**b**) Lung and solid water combination. (**c**) PMMA wedge and solid water combination. (**d**) Rando head phantom.

All phantoms were scanned with a SOMATOM Definition AS (Siemens Medical Systems, Erlangen, Germany) X-ray computerized tomography (XCT) unit. The scanning parameters used were as follows: 500 mm diameter field of view, 120 kVp, helical mode, and a slice spacing of 2 mm. The XCT scans were then imported into a commercial treatment planning system (TPS) (Syngo, Siemens, Erlangen, Germany) where the nominal plans were designed and optimized. The targets in each phantom were designed according to the characteristics of each different phantom combination. The target for the bone phantom, seen in Figure 2a, was a cuboid shape located near the central region of the combination phantom. The target overlapped with part of the bone slab and some of the solid water slabs. The target of the lung phantom, seen in Figure 2b, was similar to that of the bone phantom. The target of the wedge-shaped composite phantom, as seen in Figure 2c, was also cuboid. With a wedge angle of 30°, the water-equivalent depth changes significantly with off-axis distance and absolute penetration errors due to the XCT number to stopping power conversion errors become greater with increasing wedge thickness. The target of the head phantom, seen in Figure 2d, was drawn as a cylinder, with the axis of the cylinder parallel to the long axis of the body. The size of the cylinder was congruent with a removable muscle-equivalent cylinder placed within the brain volume of the head phantom. The treatment plans for all four phantoms utilized a horizontally directed beam with a prescribed biologically weighted dose to the targets of 2 Gy or a physical dose of 1.82 Gy.

After the dose distributions were calculated under nominal conditions in Syngo, the plans were exported, transferred, and then imported into the *in-house*-developed Treatment Information, Management, and Planning System (TIMPS^®^) to re-calculate the dose distributions under various error scenarios similar to the ranges of the typically applied uncertainties [20]. TIMPS calculates the dose using a track-repeating type of Monte Carlo-based algorithm [21,22,23]. When simulating the effects of the setup uncertainty, the DICOM coordinate system was used with shifts in the anterior-to-posterior (A-P) direction being defined as up and down and shifts in the head-to-feet (H-F) direction being defined as left and right. The lateral setup shifts were simulated in the calculations by moving the location of the portal isocenter within the patient by 3 mm in each of the four directions: up, down, left, and right. This shift distance is a commonly used 2σ lateral alignment uncertainty value for planning head and neck patients. For each of the 4 shifted directions, under-penetration errors were simulated by increasing the XCT number of the voxels within the phantom by 3.5% and decreasing the beam energy by the equivalent of 2 mm of water. Over-penetration errors were similarly simulated by decreasing the XCT number of the voxels by 3.5% and increasing the energy by the equivalent of 2 mm of water. These values are common penetration uncertainty values, again at the 2σ level, for anywhere in a patient where imaging artifacts are absent. This procedure resulted in a total of nine dose distributions (1 nominal plus 8 error scenarios).

### 2.2. DVH Analysis

The dose–volume histogram (DVH) is one of the most commonly used tools for the quality assessment of clinical treatment planning. Trofimov et al. have analyzed the effects of uncertainties by providing a family of DVHs based upon individual portal shifts in alignment and penetration [24]. In this approach, the various scenarios provide a band of DVHs, allowing visualization of the variability of the DVH under the influence of uncertainties. A narrow DVH band is desirable for the target, which would indicate that the dose distribution is relatively insensitive to uncertainties. In this study, a band of DVHs was generated for each phantom using the nine dose distributions.

### 2.3. Maximum and Minimum Doses

After the nine dose distributions were recalculated with TIMPS, the dose values of each voxel in each scenario dose distribution were queried using an *in-house*-developed program that utilizes the (Pydicom) routines of the Python programming language v3.8.0 [25]. Two additional dose distributions were then created, one called “maximum” and one called “minimum”, by taking the maximum dose value at each voxel location from all nine scenarios and placing it into the maximum distribution and taking the minimum dose value at each voxel location from all 9 scenarios and placing it into the minimum distribution. These two new dose distributions do not represent any deliverable dose distribution but instead represent possible upper and lower bounds of doses for each voxel under different scenarios.

### 2.4. Film Dosimetry

Film was chosen as the dosimeter to measure the dose distributions in the phantoms because: (1) The spatial resolution of film is higher than most other radiation detectors; (2) the two-dimensional nature of film can measure many parts of the dose distribution simultaneously; and (3) it can easily be placed into slab phantoms. A disadvantage of silver halide film, however, is its energy dependence. In this study that simulates the effect of uncertainties, a mix of proton energies is found at each depth in the phantoms surrounding the targets for the different error scenarios. For beams of protons, radiochromic film has been shown to have a lower energy dependence than silver halide film [26,27]. The radiochromic film, known as EBT3 (Ashland Company, Wilmington, DE, USA), requires no chemical development and was selected for this study.

All films were scanned with a VIDAR Advantage Pro scanner (Vidar, Herndon, VA, USA) and images acquired were converted to doses using the RIT Complete v. 6.4 software (Radiological Imaging Technology, Colorado Springs, CO, USA). The dose response of the film was calibrated using a single range-modulated proton portal with a minimum energy of 63 MeV and a maximum energy of 173 MeV. The portal used for film calibration was designed using Syngo and designed around a 50 mm cubical target in a water-equivalent phantom. A square field with sides of 60 mm in length was required to cover the target with a nearly uniform dose. To obtain multiple doses of 0.5, 1, 2, and 3 Gy at the center of the target, the portal MUs were rescaled. During irradiation, 50 mm square films were placed inside the water-equivalent phantom perpendicular to the beam axis. The dose to film was verified by measuring the dose delivered by the calibration portal with a Farmer-type thimble ionization chamber. The IAEA TRS-398 dosimetry protocol was used to convert the collected ion chamber charge to dose [28].

**Figure 3:**
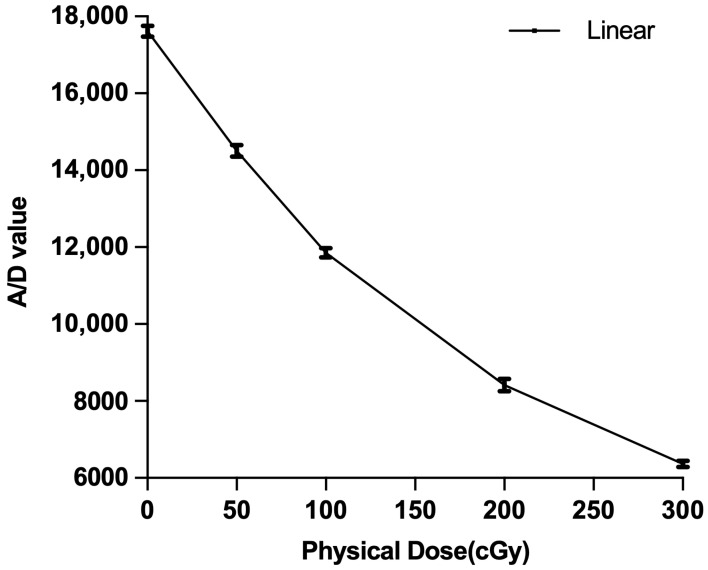
A/D value versus dose for EBT3 film exposed to the calibration portal.

After the films were exposed, RIT calibration tools were used to average the pixel values within a region of interest on each film and then construct a film response calibration curve. Figure 3 shows the resulting dose response curve. For converting the response of films exposed in the phantoms for the various error scenarios, a linear interpolation between points was performed. The vertical axis is plotted as analog-to-digital (A/D) values, which are related to the light transmitted through the film. An A/D value of 65,536 (2^16^) represents full light transmission because of the 16-bit digitization by the scanner. The lower the light transmission, the lower the A/D value. For EBT3 film, when the physical dose delivered increases, a greater proportion of radiochromic dye in the film undergoes a chemical reaction and the film darkens [29,30]. Darker films, in other words, films exposed to higher doses, thus have lower A/D values. The A/D value for a dose of zero is significantly less than 65,536 because of the background transmission through the base material and unexposed chemicals.

**Figure 4:**
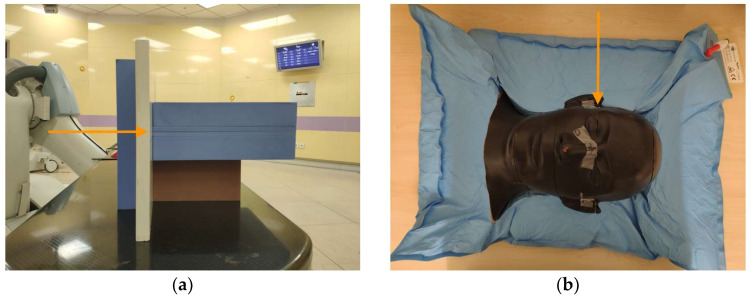
Configuration of phantoms when exposing film. (**a**) Slab phantom. (**b**) Head phantom.

Figure 4a shows the setup used for the film when irradiating the slab phantoms containing heterogeneities. The film was placed parallel to the direction of the proton beam (shown by an orange arrow), behind the entrance solid water and non-solid water test slabs, and between two large solid water slabs. In this configuration, the tunneling of protons through a gap between slabs was minimized, and the distal edge of the dose distribution could easily be determined for different off-axis locations. To facilitate the registration of the film results with the phantom plan, small marks were placed on the film according to the wall-mounted lasers, which have a daily check tolerance with respect to the isocenter of ± 1 mm. During the measurement of the simulated positioning errors, the patient positioner was moved 3 mm in each of the four directions perpendicular to the beam axis: upward, downward, left, and right. For the head phantom (see Figure 4b), the film was cut to an irregular shape before it was inserted into the phantom so that its edges matched the surface contour of the head.

### 2.5. Comparison of Measured to Calculated Profiles

**Figure 6:**
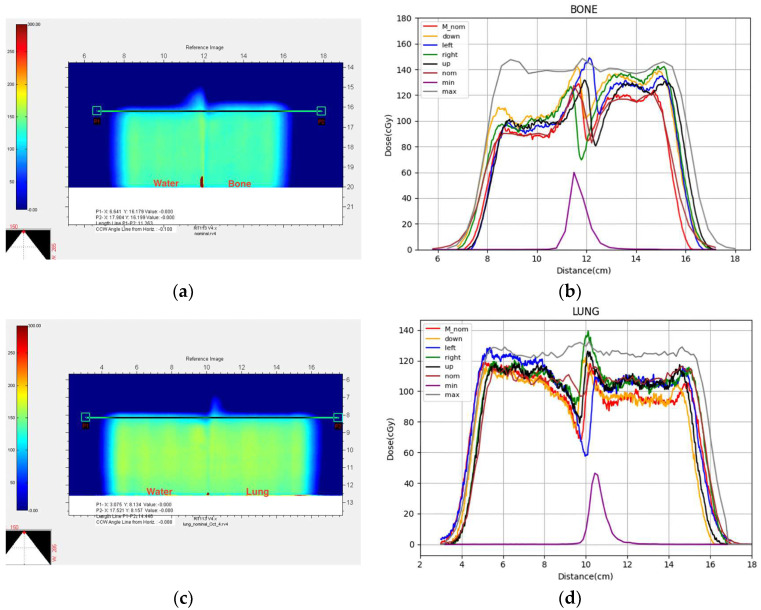

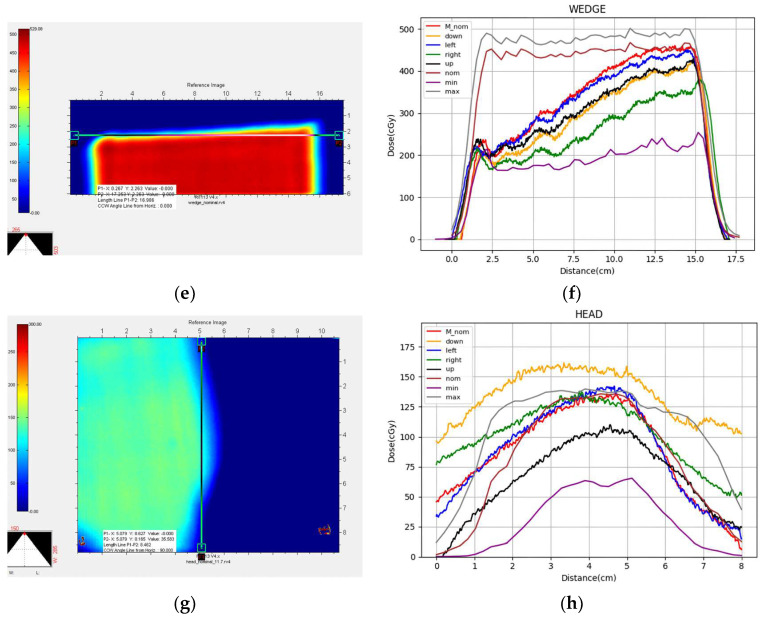
Comparison of the measured dose profiles for the nominal and four measurement shifts compared to the maximum, nominal, and minimum doses obtained from the nine-scenario calculation method. The figures on the left show the 2D film scans converted to doses while the figures on the right show the extracted dose profiles along the lines superimposed upon the film images. (**a**) Bone phantom combination, (**c**) lung phantom combination, (**e**) wedge phantom combination, and (**g**) head phantom. For Figures (**a**,**c**,**e**), the beam enters the film from the bottom of the picture, while for Figure (**g**), the beam enters the film from the left of the picture. In each Figure on the right (**b**,**d**,**f**,**h**), the red curve represents the nominal measurement condition. The orange, blue, green, and black curves represent the measured profiles of the downward, left, right, and upward 3 mm shifts in the phantom, respectively. The brown curve represents the nominal condition of the calculated plan, while the gray and purple curves, respectively, represent the maximum and minimum doses of the calculated plan.

The dose distributions measured with film were compared with the maximum and minimum voxel doses calculated from the nine scenarios. The comparison method was as follows: first, lines to evaluate the doses were selected on both the film image and in the calculated plan that were parallel to the phantom surface at a depth just proximal to the deepest penetration of the beam (see line between points P1 and P2 in Figure 6). Next, a dose scaling factor for the film was determined for the nominal scenario by taking the ratio of the calculated doses to the measured doses at several locations. This nominal scaling factor was then applied to each of the uncertainty scenarios to provide a consistent calibration.

## 3. Results

**Figure 5:**
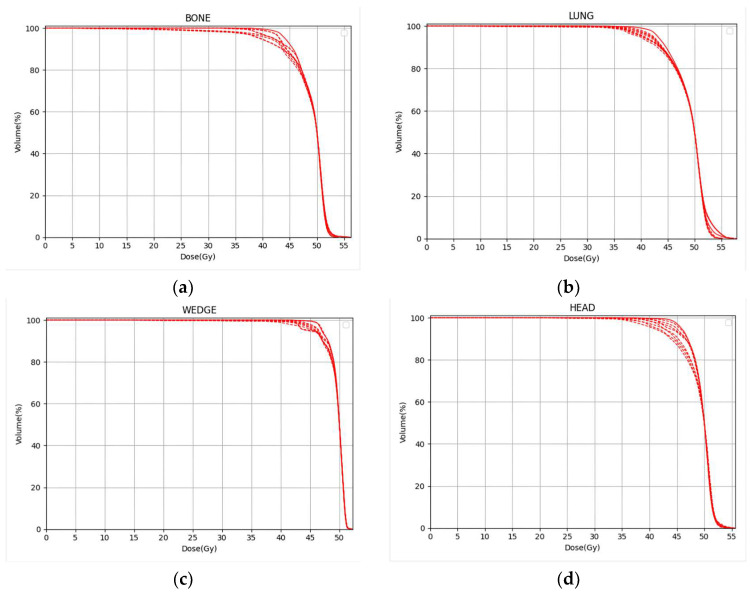
Target DVHs for all nine scenarios for each of the four phantoms. The solid curves represent the nominal doses. The dotted curves represent the other eight scenario doses. (**a**) Bone phantom. (**b**) Lung phantom. (**c**) Wedge phantom. (**d**) Head phantom.

Figure 5 plots all nine calculated scenario DVHs for the targets of each of the four phantom combinations, while Table 1 gives the range of DVH parameters derived from the DVHs. The minimum dose delivered to 100% of the targets (D_min_) is seen to decrease significantly under the influence of uncertainties, with the volume of the target covered by 95% of the prescribed dose (V_95%_) also decreasing. This is expected because no margins for uncertainties were applied around the targets. In contrast, the uncertainties have little effect on the maximum dose delivered to the target (D_max_). On the other hand, D_max_ would be expected to increase for OARs just adjacent to the target.

Figure 6 shows, on the left side, images of the film scans after conversion to dose and, on the right side, the dose profiles extracted from each film for each shift. For the bone and lung phantoms immediately downstream of the interface between the two materials, the dose distribution bulges inwards and outwards on opposite sides of the projection of the interface plane. The dose within the bulging region for all nine scenarios is between the maximum and minimum predicted (calculated) doses for almost all points along the profile. Outside of the bulging region, the measurement shows that the depth to the distal edge of the dose distribution is somewhat variable with off-axis position in contrast to the plan where the depth of the distal edge is constant. The measured doses for all nine scenarios are, however, between the maximum and minimum predicted doses. In the bone phantom combination, the dose along the profile behind the bone half of the phantom is larger than beyond the water side of the phantom. For the lung phantom combination, the dose along the profile behind the lung half of the phantom is slightly smaller than beyond the water side of the phantom. For the wedge-shaped phantom, all five measured scenarios, including the nominal case, have trapezoidal-shaped dose distributions, whereas the calculated dose distributions are almost flat. The dose along the profile is smaller behind thicker parts of the wedge, indicating a shorter penetration than calculated. All of the dose profiles for the head phantom are parabolic in shape. For the scenario where the phantom is shifted downwards with respect to the beam (yellow curve), the measured dose exceeds the maximum calculated dose at most off-axis positions.

A detailed comparison of the measured and calculated dose profiles at the distal edge for the bone phantom showed that all measured doses for all scenarios were between the maximum and minimum calculated doses, except for a small region of the left displacement condition. Near the peak of this profile (shown in blue), the measured dose was 147.38 cGy, which is only 0.72 cGy more than the peak dose of the maximum calculated dose (shown in gray). For the lung phantom, like the bone phantom, all measured doses for all scenarios were between the maximum and minimum calculated doses, except for a small region of the right displacement condition. This region also occurred at the peak position, with the measured dose in this case being 6.33 cGy larger than the calculated maximum dose. For the wedge phantom, the right-displaced measurement profile was shifted slightly outside of the maximum calculated dose profile by about one voxel. At the extreme left side of the field (from 1.3 cm to 2.6 cm position) where the wedge is thickest, several of the measured dose profiles were lower than the minimum calculated dose. For the head phantom, almost the entire profile for the downward displaced situation (orange curve) was above the maximum calculated dose profile (shown in gray). At the periphery of the field on both sides, several other measured profiles presented greater doses than the maximum calculated dose profile. None of the measured profiles gave a smaller dose than the minimum calculated dose profile.

## 4. Discussion

The film response to the dose calibration function used a linear interpolation method, resulting in small dose errors in the measured dose profiles. For example, a pixel with a measured A/D of 10,000 would convert to a dose of 150 cGy. In fact, the dose may have been slightly lower at the A/D value of 10,000. A calibration curve with more measured doses should provide a more accurate dose conversion but, unfortunately, substantially more accelerator beam time is required.

Inside the bone phantom, a hill and valley were seen in both the measured and calculated dose profiles just distal to the bone/solid water interface. This phenomenon is caused by the scattering of protons when the densities of the two adjacent media are greatly different—this phenomenon is called an “interface effect” [31]. In order for the proton beam to pass through the bone and water slabs and stop at the same physical thickness near the distal edge of the target, a higher energy is required for the part of the beam passing through the bone region than is required for the part of the beam passing through the solid water region. Some of the higher energy protons entering the bone side of the phantom, however, scatter into the solid water region of the phantom but then exit the distal side of the target with a slightly higher residual energy. Similarly, some of the lower energy protons entering through the solid water side of the phantom scatter into the bone region of the phantom but then run out of energy and stop before exiting the target. This causes too large of a dose (the hill) behind the water side but too small of a dose (the valley) behind the bone side. In the figure comparing the measured values with the calculated values, the expected situation is that the position of the peak value of the profile should be displaced from the nominal position at the same distance as the phantom was displaced in the left and right directions, i.e., ±3 mm. In the case of the downward and upward phantom displacements perpendicular to the film plane, the peak position of the profile on the film should not change. In the actual situation, however, errors in the irradiation process and errors in the extraction of the dose values can lead to deviations from the expected situation but still be within the 3 mm displacements. Figure 6b, therefore, shows a band of profile curves representing the uncertainty distribution.

The lung phantom also presented a case where two different media were adjacent, and a profile with a hill and valley behind the target region was formed. In this case, the hill was behind the solid water region, and the valley was behind the lung region. Compared with the bone phantom results, the actual measurement of the lung phantom had less deviations and was closer to the expected situation.

For the wedge phantom, the shape of the dose profiles of the measured and calculated values along the yellow reference line did not match. One explanation could be that the XCT number to relative linear stopping power is not properly converted by the standard tissue conversion function. Another possible explanation could be that, although the phantom and target were perfectly aligned in the TPS plan, and the optimization algorithm was able to achieve a uniform dose across the distal edge of the target, during the actual irradiation process, the wedge may have been setup slightly rotated with respect to the planned orientation, thus changing the effective thickness of the wedge. In the case of a wedge rotation error combined with a 3 mm displacement, near the thick end of the wedge, the beam would pass through a thicker part of the wedge compared to the plan, and thus, the penetration of the beam in the phantom would be slightly less, giving a lower dose. Near the thin end of the wedge, there would be very little difference in the water-equivalent thickness if the wedge was rotated or displaced, and thus, the measured value of 447 cGy was nearly the same as the planned nominal dose. For downwards and upwards displacements, represented by the orange and black curves, the shape was nearly the same as the left and right displacements, indicating that most of the dose deviations come from the wedge rotation rather than displacement. Both possible explanations are conditions as to why an uncertainty evaluation is needed during planning.

In the comparison between the measured and calculated profiles of the head phantom (seen in Figure 6h), the most obvious differences are for the upwards (orange) and downwards (black) displacements. Unlike the other phantoms, where the water equivalent thickness changes in only one direction, the surface of the head phantom changes in all directions. In the head plan, the ear was in the middle of the field, which, for small changes in lateral displacement, could cause large changes in the penetration. Similarly to the dose profiles in the bone and lung phantoms behind the material interface, the calculated maximum possible doses behind the ear could be slightly underestimated. Robust planning techniques are typically used to reduce the effect of large gradients and should be used in this situation. Other possibilities for the observed dose deviations are a change in the film position or depth within the phantom or a change in the air gap thickness between the phantom slabs to accommodate the film. Neither of these uncertainties would be present in a patient treatment.

A major limitation of this study was that only four phantoms and target shapes were used and therefore do not cover all possible patient conditions. On the other hand, the selected phantoms and targets did utilize a variety of shapes (flat, steep wedged, and curved) and a wide range of densities that are encountered in real patients. Another limitation was that intentional rotations were not tested, although small rotations may have occurred during the alignment of the wedge and head phantoms. Nevertheless, this rare comparison of measurements to calculations with intentional errors provided some interesting data.

As was noted in Section 3, there were a very small number of datapoints where the measured doses were outside of the bounds of the maximum and minimum doses from the uncertainty calculations. The uncertainty ranges chosen for this study were based upon 2σ (95% confidence level) of the expected uncertainties typically used in patient planning and delivery. Some people have advocated for only taking into account 1.5 σ (87% confidence level) of the uncertainties, because if one tries to account for 100% of all uncertainties, it often might not be feasible to treat the patient while achieving all of the dose constraints [32]. In addition, for most treatments, daily doses would be found between the minimum and maximum possible calculated doses, perhaps randomly distributed or perhaps systematically distributed near one extreme. Either way, a majority of the errors would be caught by a nine-scenario approach. Increasing the number of calculated scenarios might result in finding a few more points outside of the minimum and maximum bounds but the calculation time might become prohibitively long for only small gains in detection. Sterpine et al. mentioned that using 20 and 80 scenarios gave similar results [11]. Whether there is much difference between 9 and 20 scenarios has not yet been studied.

As evidenced by these experimental findings, uncertainties in planning and delivery can significantly impact clinical results, potentially causing OAR damage and diminished therapeutic efficacy. Consequently, the pre-treatment uncertainty evaluation is essential prior to implementing a plan in the clinic.

## 5. Conclusions

In this study that compared the actual measured doses from various uncertainty scenarios with the minimum and maximum doses of a calculated multi-scenario method, it was shown that the calculation method can, using nine scenarios and estimated uncertainty values, capture most possible doses. There were a few points in some scenarios, however, where the measured doses were slightly outside of the bounds of the minimum or maximum calculated doses. The applied alignment shifts and penetration uncertainties were chosen to represent 2σ of the expected uncertainties typically used in patient planning and delivery. This means that the actual delivered voxel doses should be between the maximum and minimum doses 95% of the time; this level appears to have been achieved. The nine-scenario method is thus a reasonable method for effectively evaluating the robustness of simple mono-directional plans containing heterogeneities. Calculating more different scenarios or increasing the value of uncertainty parameters may increase the probability that the envelope bounded by the maximum and minimum calculated doses contains all the measurements. Future work should expand this investigation to include multi-portal optimized plans, plans with patient motion, and OAR doses.

## Figures and Tables

**Table 1:** Range of DVH parameters from the nine different scenarios for the target volume in each of the four phantoms.

	D m i n	D m a x	V_95%_
Bone	4.28 Gy–37.94 Gy	52.46 Gy–56.34 Gy	86.07–98.60%
Lung	4.20 Gy–38.82 Gy	55.16 Gy–57.83 Gy	85.49–98.80%
Wedge	11.83 Gy–41.90 Gy	51.96 Gy–52.27 Gy	97.63–99.68%
Head	19.56 Gy–37.33 Gy	53.93 Gy–55.62 Gy	85.77–99.06%

## Data Availability

The original data presented in the study are openly available in [Raw Data_Comparison_multi-scenario_measurement] at [10.5281/zenodo.15742631].

## Abbreviations

The following abbreviations are used in this manuscript:

D_min_	the minimum dose delivered to 100% of the specified volume of interest (also known as D_100%_)
D_max_	the maximum dose delivered to any part of the volume of interest (also known as D_0%_)
D_RBE_	RBE weighted dose
DVH	dose volume histogram
LET	linear energy transfer
OAR	organ at risk
PMMA	polymethylmethacrylate
PTV	planning target volume
RBE	relative biological effectiveness
TPS	treatment planning system
V_95_	the volume receiving 95% of the specified dose
XCT	X ray computed tomography
